# Crystal structure of the antimicrobial peptidase lysostaphin from *Staphylococcus simulans*

**DOI:** 10.1111/febs.12929

**Published:** 2014-08-01

**Authors:** Izabela Sabala, Elzbieta Jagielska, Philip T Bardelang, Honorata Czapinska, Sven O Dahms, Jason A Sharpe, Richard James, Manuel E Than, Neil R Thomas, Matthias Bochtler

**Affiliations:** 1 International Institute of Molecular and Cell BiologyWarsaw, Poland; 2 Centre for Biomolecular Sciences, School of Chemistry, The University of NottinghamUK; 3 Centre for Biomolecular Sciences, School of Molecular Medical Sciences, The University of NottinghamUK; 4 Leibniz Institute for Age Research – Fritz Lipmann Institute (FLI)Jena, Germany; 5 Institute of Biochemistry and Biophysics, Polish Academy of SciencesWarsaw, Poland

**Keywords:** crystal structure, lysis, lysostaphin, *Staphylococcus aureus*, *Staphylococcus simulans*

## Abstract

*Staphylococcus simulans* biovar *staphylolyticus* lysostaphin efficiently cleaves *Staphylococcus aureus* cell walls. The protein is in late clinical trials as a topical anti-staphylococcal agent, and can be used to prevent staphylococcal growth on artificial surfaces. Moreover, the gene has been both stably engineered into and virally delivered to mice or livestock to obtain resistance against staphylococci. Here, we report the first crystal structure of mature lysostaphin and two structures of its isolated catalytic domain at 3.5, 1.78 and 1.26 Å resolution, respectively. The structure of the mature active enzyme confirms its expected organization into catalytic and cell-wall-targeting domains. It also indicates that the domains are mobile with respect to each other because of the presence of a highly flexible peptide linker. The high-resolution structures of the catalytic domain provide details of Zn^2+^ coordination and may serve as a starting point for the engineering of lysostaphin variants with improved biotechnological characteristics.

**Structured digital abstract:**

- lysostaphin by x-ray crystallography (1,2).

## Introduction

*Staphylococcus aureus* is a common human and animal pathogen of major clinical significance 1. *Staphylococcus simulans* lysostaphin (EC 3.4.24.75) has staphylolytic properties that were originally discovered in the 1960s 2,3 and immediately attracted interest as a means of combating staphylococcal infection 4. The topical or systemic use of lysostaphin has since proven effective in several mouse and rat models of staphylococcal infection 5,6. Moreover, transgenic mice and cattle producing the enzyme have been generated to engineer *S. aureus* and/or *S. epidermidis* resistance 7,8, and adenovirus-mediated lysostaphin delivery has been tested in goats 9,10. In humans, the protein has been effective in the experimental treatment of *S. aureus*-infected patients 11, and is currently in clinical trials for use in treating burns 12. Lysostaphin has also shown promise for the elimination of *S. aureus* in infected asymptomatic carriers 13, which might be useful in a hospital setting. Apart from direct application in humans, lysostaphin has also been considered for the eradication of *S. aureus* biofilms from artificial surfaces 14 and as an antimicrobial agent in catheter coatings 15.

**Fig 1:**
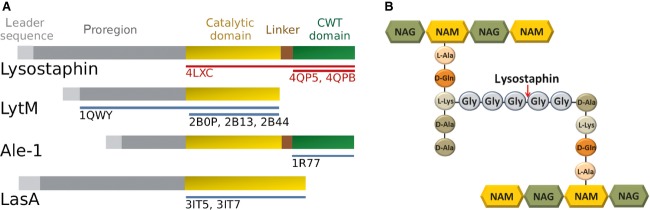
Domain organization of lysostaphin and related enzymes. Blue and red bars (labelled by PDB accession codes) indicate fragments structurally characterized earlier and in this work, respectively. Gray colors are used for regions of unknown structure.

Lysostaphin is produced as a preproprotein (UniProt entry P10547) with a leader sequence (residues 1–23), inhibitory proregion (residues 24–247) 16, catalytic domain (residues 248–384), linker (residues 385–400) and cell-wall-targeting (CWT) domain (residues 401–493, UniProt numbering used throughout) (Fig. 1A). The latter three constitute the mature peptidase, henceforth termed ‘lysostaphin’. This cuts the peptide bond between the third and fourth glycine residues of the pentaglycine cross-link in the peptidoglycan (Fig. 1B) 17. The active enzyme shares structurally characterized domains with LytM 18,19, Ale-1 20 and LasA 21, but only the structure of mature lysostaphin can shed light on crucial aspects of the domain arrangement or substrate binding of this biotechnologically important enzyme.

Lysostaphin has many favorable properties (e.g. dependence of the enzyme activity on the ionic milieu compatible with *in vivo* applications) that are not shared by some of its homologues 22. However, widespread use of lysostaphin has also identified its limitations. *Staphylococcus aureus* cells can acquire lysostaphin resistance by altering cross-bridges between peptidoglycan stem peptides 23. A structure of lysostaphin might, therefore, be helpful in understanding its favorable properties, which could then be grafted onto homologues that do not share them (such as LytM 22), as well as being used to generate variants of lysostaphin that overcome the limitations of the natural enzyme.

A crystal structure of lysostaphin has not yet been reported, despite widespread interest in the protein and the possible applicability of structural information. Here, we present a low-resolution (3.5 Å) structure of mature lysostaphin with catalytic and CWT domains, and two high-resolution (1.26 and 1.78 Å) crystal structures of the catalytic domain in isolation, which elucidate details of the active site architecture.

## Results and discussion

### Crystallization and structure determination of mature lysostaphin

**Fig 2:**
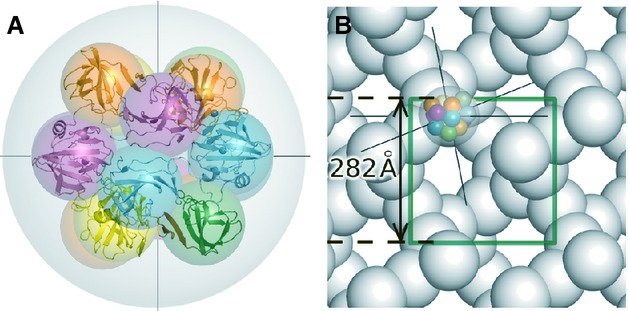
Crystal structure of mature lysostaphin. (A) Four crystallographically independent lysostaphin molecules (ribbon models) and their symmetry mates (behind them) form a hollow ‘ball’ of 222 point symmetry composed of altogether eight lysostaphin molecules (gray). The twofold symmetry axes are indicated as lines. (B) Packing of the ‘balls’ in (A) into the crystal lattice. The ‘face’ of the ball presented in (A) is turned towards the lower right-hand corner in (B).

Several differently tagged variants of mature lysostaphin (with catalytic and CWT domains, but without the proregion and leader peptide) were produced in the ectopic host *Escherichia coli* and purified by affinity chromatography and gel-filtration steps. The protein proved difficult to crystallize. Altogether, over 10 000 possible conditions were screened until diffracting crystals were obtained. Crystals diffracted to 3.5 Å and belonged to space group P4(3)32 with a large unit cell (282 Å length). Nevertheless, they contained only four molecules of lysostaphin in the asymmetric unit, which corresponds to > 85% solvent content (observed for only 0.05% of other crystals in the PDB). In retrospect, the large solvent content can be attributed to a highly unusual crystal packing. Although lysostaphin is a monomer in solution 24, pairs of lysostaphin molecules (adopting different conformations) assemble to form ‘heterodimers’. Because of a combination of local and crystallographic twofold symmetry, the original ‘heterodimers’ in turn form hollow, ball-like structures with 222-point symmetry, built of eight lysostaphin molecules (Fig. 2A). Although balls can, in principle, be packed very tightly (74% of space filled 25), the assembly of balls in the lysostaphin crystals is characterized by large pores that are clearly visible in projections along the unit cell axes (Fig. 2B) and along the body diagonals (not shown). The phase problem for crystals of mature lysostaphin was solved using a combination of molecular replacement (search models based on deposited crystal structures of catalytic domain of LytM and CWT domain of Ale-1, respectively) and multiple anomalous diffraction (MAD) approaches. Data collection and refinement parameters are presented in Table 1.

**Table 1:** Data collection and refinement statistics

	Data collection statistics				
	Full-length lysostaphin			Catalytic domain	
	MAD peak	MAD IP	Native^a^		
Space group	P4(3)32	P4(3)32	P4(3)32	P2(1)	P2(1)
*Cell dimensions*					
*a* (Å)	283.6	283.3	282.0	34.3	34.3
*b* (Å)				106.8	107.3
*c* (Å)				34.3	34.3
β (°)				97.4	97.6
Beamline	DIAMOND IO2		BESSY 14.1	BESSY 14.1	BESSY 14.2
Wavelength (Å)	1.2827	1.2832	0.9184	1.2000	0.9184
Resolution (Å)	35–4.0	35–4.0	50–3.5	50–1.26	35–1.78
Lowest shell	35–17.85	35–17.84	50–15.65	50–3.76	35–5.28
Highest shell	4.09–3.99	4.09–3.99	3.59–3.5	1.33–1.26	1.89–1.78
*R*_sym_ (%)^b^	28.5 (6.5, 54.2)	14.7 (3.2, 49.0)	29.5 (5.1, 90.8)	7.2 (5.6, 41.2)	16.7 (5.7, 58.6)
*R*_meas_ (%)^b^	34.2 (7.8, 65.7)	17.6 (3.8, 58.9)	30.0 (5.2, 92.0)	7.8 (6.1, 46.6)	21.3 (7.3, 73.0)
*CC*_1/2_^b^	93.8 (98.8, 77.7)	98.2 (99.8, 82.3)	99.6 (100, 94.4)	99.8 (99.7, 84.8)	96.6 (99.1, 68.6)
*I*/σ*I*^b^	5.6 (12.1, 2.8)	8.0 (18.5, 3.5)	17.3 (57.0, 5.3)	13.2 (30.8, 2.5)	7.5 (17.0, 2.1)
Completeness (%)^b^	96.3 (82.6, 97.8)	98.7 (82.3, 98.7)	100.0 (100, 95.5)	98.3 (99.8, 90.9)	99.1 (97.1, 98.8)
Multiplicity^b^	3.17 (3.30, 3.11)	3.22 (3.29, 3.22)	36.4 (28.9, 37.9)	5.8 (6.3, 4.5)	2.7 (2.7, 2.7)
V_M_			8.3	2.0	2.0
Solvent content (%)			85.2	39.6	39.9
Twin law				l,-k,h	(l,-k,h)^d^
Twin fraction (%)					
Estimated				37	22
Refined				36.0	–
<|E^2^ − 1|>	0.758	0.776	0.746	0.622	0.700
<|L|>, <L^2^>	0.468, 0.296	0.493, 0.323	0.488, 0.317	0.409, 0.232	0.459, 0.283
*Refinement statistics*					
Resolution			50–3.5	35–1.26	35–1.78
Highest shell (Å)			3.59–3.50	1.30–1.27^f^	1.88–1.78^f^
No. reflections			48 689	64 902	23 389
*R*_work/_*R*_free_^c^			26.8/28.7	13.0/17.5	21.0/24.4
Highest shell			30.4/35.6	20.9/30.7	32.1/33.3
No. atoms^e^			7589	2495 (2328)	2378 (2325)
Protein			7540	2251 (2086)	2135 (2082)
Ligand/ion			49	40	6
Solvent			0	220 (218)	237
Rmsd					
Bond lengths (Å)			0.009	0.010	0.023
Bond angles (°)			1.2	1.2	1.8
Ramachandran					
Allowed region (%)			100.0	100.0	100.0
Favored region (%)			93.56	97.5	97.4
Molprobity clashscore			6	3.6	4.1

a The native data contain Zn^2+^ anomalous scatterers, but have been collected at a wavelength remote from the absorption peak.

b Data for the lowest and highest resolution shells are shown in parentheses. The completeness for the data for the lowest shell and full resolution is calculated versus the number of possible reflections from infinity to the stated high-resolution limit.

c Data for the highest resolution shells are shown in parentheses.

d The twin component was not substantial enough to be used in the refinement (not to reduce the data to parameter ratio).

e Alternative conformations counted separately (the true number of atoms is shown in parentheses).

f Shells adjusted so that they include an R_free_ reflection shell.

Because multiple attempts to improve the diffraction limit of the mature lysostaphin crystals at synchrotrons (using annealing, dehydration, etc.) proved unsuccessful, we produced the catalytic domain in isolation, hoping that the globular nature of the domain would facilitate crystal packing. Indeed, crystals in space group P2(1) could be grown at similar pH in the presence and absence of phosphate. They diffracted to 1.26 and 1.78 Å resolution, respectively. The strongest diffracting crystals were pseudomerohedrally twinned 26 with a twofold twinning axis along a face diagonal, made possible by the ‘coincidental’ equality of unit cell constants. Both structures could be readily solved by molecular replacement. For refinement, test reflections were chosen in thin-resolution shells to avoid introducing correlations between reflections. Data collection and refinement statistics for both structures are summarized in Table 1.

### Overall lysostaphin structure

**Fig 3:**
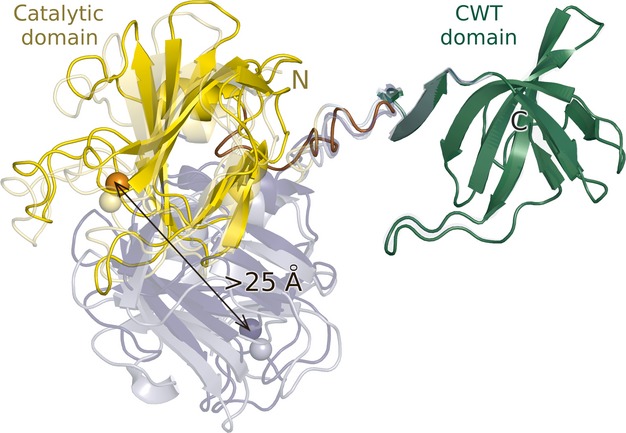
Domain mobility in full-length lysostaphin. Molecules in the asymmetric unit of the crystal are shown in ribbon representation, with the catalytic zinc ion shown as a ball. CWT (green) domains were superimposed. Catalytic domains (dark/light yellow and blue) differ in orientation by almost 100°.

**Fig 4:**
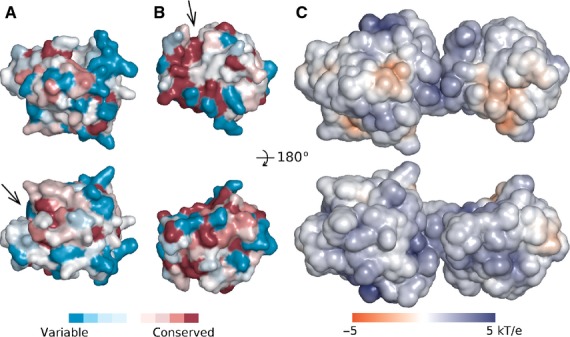
Sequence conservation and electrostatics of lysostaphin. The solvent-excluded surface of (A) catalytic and (B) CWT lysostaphin domain was colored according to the sequence conservation determined using the Consurf server 55, with a set of sequences selected for analogous function and possibly diverse sequence (UniProt codes: H0DEX1, Q4L983, D3X7N2, F0P463, I7IJS5, I7JXS6, O05156, J9GY33, J9GX22, D6A2X0, Q99WV0, Q99X10, G5JF40, K9AV95, K0TTI9, K0UDK3 for catalytic domain; and D3X7N2, Q99WV0, O05156, H0DJE6, B9CV69, D3QFD1, J0GU31, H3UFQ3, D4FI37, Q4L3Y5, H9A141, F9L8Y9, E5CSC2, O56788 for the CWT domain). (C) Solvent-accessible surface of lysostaphin colored according to the electrostatic potential calculated with the delphi program 56. The orientation in the top row is rotated 90° with respect the yellow/green molecule in Fig. 3 (and similar to Fig. 7A), the location of the putative substrate-binding grooves is marked by arrows.

The crystal structure shows the expected overall organization of lysostaphin into an N-terminal catalytic domain and a C-terminal CWT domain (Fig. 3). When superimposed separately, the domains of the four molecules in the asymmetric unit of the mature lysostaphin crystals overlap very well, which may in part reflect the use of noncrystallographic symmetry restraints necessary at this resolution. However, we also observe essentially the same conformation in the crystals of the catalytic domain in isolation, suggesting that, at least, the structure of this domain is robust to changes in buffer composition. Amino acid conservation scores among selected sequences of catalytic and CWT domains from various *Staphylococci* and their bacteriophages, showing up to 45% sequence identity to lysostaphin were mapped to the surface of its domains and are high in the region of the active site, and also in the likely peptide-binding region of the CWT domain (Fig. 4A,B). The electrostatic surface of lysostaphin is a meshwork of regions of positive and negative potential, without large hydrophobic regions, consistent with either solvent exposure or binding to the pentaglycine peptide (containing multiple hydrogen bond donors and acceptors) (Fig. 4C).

### Catalytic domain

**Fig 5:**
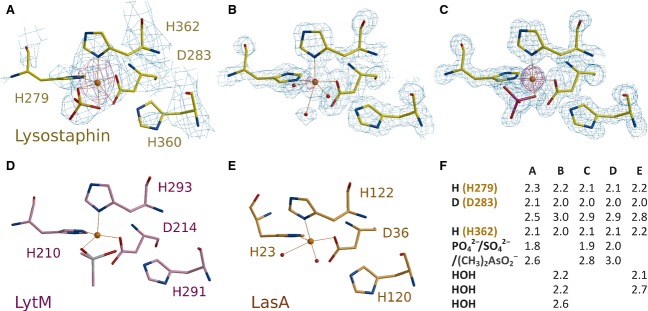
Catalytic sites of lysostaphin and related enzymes. (A) Active sites in the crystals of mature lysostaphin, the catalytic domain crystals were grown in either the absence (B) or presence (C) of phosphate; (D) active LytM and (E) LasA (previously characterized closely related peptidases). (F) Metal–ligand distances (in Å) for (A–E). The low resolution makes interpretation of the density in (A) very tentative. The distances shown in (E) differ between molecules in the asymmetric unit for all presented structures. The electron-density maps shown in cyan in are composite omit maps contoured at 1.5 rmsd. The maps shown in magenta are Bijvoet-difference Fourier (anomalous scattering density) maps calculated with the phases from the final models depleted of Zn^2+^ ions, and contoured at 6 rmsd.

The lysostaphin catalytic domain shares key features of the M23 family peptidases 27. The core of these structures is an antiparallel β sheet that anchors the catalytic residues, which are grouped around a central Zn^2+^ cation (Fig. 5). Identification of this metal ion was confirmed crystallographically (by a drop in the anomalous density peak height at the metal ion sites passing from the absorption to the inflection point wavelength). In the high-resolution structures it is additionally supported by typical metal–ligand distances and similar temperature factors for the metal ion and the surrounding ligands. The Zn^2+^ ion is coordinated by His279 and Asp283 of the characteristic HxxxD motif and His362 of the HxH motif of LAS peptidases, as expected.

**Fig 6:**
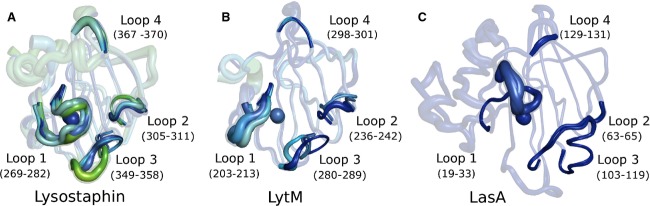
Lysostaphin, LytM and LasA active site clefts. Internal variability of the loops surrounding the active sites of (A) lysostaphin, (B) LytM and (C) LasA as captured by multiple molecules in the asymmetric units of their different crystal forms. The numbering of the loops follows the nomenclature of Spencer *et al*. 21 The diameter of the ‘tubes’ and the color scheme correspond to the magnitude of the residual temperature factors ranging from blue (low) to red (high).

The active site is located in a substrate-binding groove, as in other MEROPS M23 family proteins. The floor of the groove is built by the β sheet of the protein and the walls are made up of loops L1–L4 as defined previously for LasA 21 and LytM 19. The long L-shaped loop 1 is located above the Zn^2+^ ion forming a roof above the active site oriented towards the middle of the groove. This loop and loops 3 and 4 have fairly similar conformations in all lysostaphin molecules in the three crystal forms. By contrast, the smaller loop 2 seems to be the most flexible (Fig. 6).

Zn^2+^ coordination cannot be deduced with confidence for the mature lysostaphin structure, because of the limited resolution. Metal–ligand distance restrictions had to be imposed so that the zinc density does not dominate the positions of the lighter atoms. In one subunit, we tentatively modeled a sulfate ion (from the buffer) directly coordinating the Zn^2+^ ion. By contrast, the structures of the catalytic domain are very informative in this respect. In crystals grown in the absence of phosphate ions, we observed a hexa-coordinated Zn^2+^ ion in the active site, with the expected three amino acid ligands (His279, Asp283 and His362) and three water molecules, in a nearly perfect octahedral arrangement. In crystals grown in the presence of phosphate, the Zn^2+^ amino acid ligands are arranged similarly, but instead of the water molecules, a phosphate ion is directly coordinated to the Zn^2+^ ion. Two of the phosphate oxygen atoms are roughly in the positions previously occupied by water molecules (although the oxygen–oxygen distance is shorter for the phosphate than for the two water molecule oxygens). Such coordination is not unusual for Zn^2+^ coordination by bidentate ligand atoms 28, and has also been described for LytM 19 (Fig. 5).

Tetracoordination of the Zn^2+^ ion is typical for most LAS enzyme crystal structures, but the Zn^2+^ ion in LasA from *P. aeruginosa* is pentacoordinated 21. Pentacoordination is consistent with many proposals for catalytic mechanism, and also with EPR data for a Co^2+^-substituted VanX (an enzyme that belongs to the LAS superfamily, but not to metallopeptidase clan M23) 29. To our knowledge, hexacoordination with only amino acid and water (hydroxide) ligands has so far not been observed for any other LAS enzyme. Differences in coordination state may reflect variations between LAS family members, but more likely they simply reflect the low energy barriers for changes of Zn^2+^ coordination 30, which may also play a role in catalysis 31.

### CWT domain

**Fig 7:**
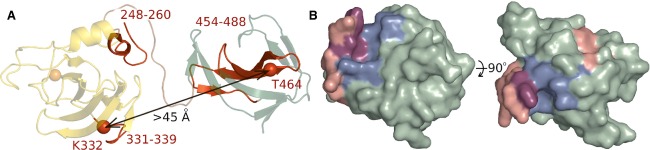
Mapping of residues implicated in interdomain contact and peptidoglycan binding onto the lysostaphin structure. (A) Full-length lysostaphin structure in ribbon representation. Peptides that show a hydrogen/deuterium effect because of domain interaction are highlighted in red. The Cα atoms of residues that were replaced with cysteines and were cross-linked are shown as red balls 12. The catalytic and CWT domains are shown in yellow and green, the flexible linker is in brown. (B) Surface representation of lysostaphin CWT domain. Lysostaphin counterparts of residues identified as responsible for substrate binding in Ale-1 by Lu *et al*. 20 and Hirakawa *et al*. 34 are shown in pink and blue, the residues identified by both studies are shown in magenta. The orientation of the CWT domain in the left-hand panel of (B) is analogous to that in (A).

The CWT domain of lysostaphin is highly similar in sequence (> 80% identity) and structure to the CWT of Ale-1 20 (main chain atom rmsd 0.5 Å). Biochemical and structural studies indicate that this domain interacts directly with interpeptide bridges present in peptidoglycans 32. The fold classifies the domain as SH3b, the prokaryotic counterpart of the eukaryotic SH3 domain 33. At present, structural information on how the lysostaphin/Ale-1 family of CWT domains interacts with substrates is rather limited, as neither this work nor prior experiments with Ale-1 succeeded in crystallizing a CWT with bound substrate. Nevertheless, some informed speculation is possible. Based on a combination of structural studies, site-directed mutagenesis experiments and computational docking, amino acid residues of Ale-1 that affect peptidoglycan binding have been identified 20,34. Because of the high sequence and structural similarity between the lysostaphin and Ale-1 CWT domains, the equivalent amino acids in the lysostaphin structure can be readily identified and located. Together, they indicate a relatively wide peptidoglycan-binding site, with notable enrichment of binding-related residues in the central groove of the domain (Fig. 7).

### Different linker conformations, domain mobility

Comparison of the four lysostaphin molecules in the asymmetric unit of the crystals using the DynDom server 35 shows that they can be divided into pairs of molecules of very similar overall structure. However, between the pairs, there is major variation in the relative domain orientation, because of differences in the linker conformation around Gly388. The local differences in the linkers have drastic overall consequences. When CWT domains are superimposed, the catalytic domain orientations differ by ∼ 100° and active sites are up to ∼ 25 Å from each other (Fig. 3). The linker amino acid composition in lysostaphin (rich in glycines) classifies the linker as flexible 36. Moreover, analysis of the amino acid conservation among lysostaphin and those homologues that share the architecture of N-terminal catalytic and C-terminal CWT domain reveals that the linker is among the least conserved regions of the proteins (conservation is even lower in the proregions).

Biochemical data suggest that domain mobility in solution exceeds the range observed in the crystal structure. Experimentally introduced cysteines in positions 332 (K332C) and 464 (T464C) can be cross-linked under oxidizing conditions 12, but the Cα carbon atoms of the mutated residues are > 45 Å apart in all four molecules in the structure and cannot even be approximated by a simple closure or hinge between lysostaphin domains (Fig. 7A).

Possible interactions between the catalytic and CWT domains of lysostaphin were previously studied by comparing exposure to hydrogen/deuterium exchange between the full-length protein and its isolated domains 12. In light of the structural data, the reported greater exposure of peptide 248–260 for the isolated domains is expected, because these residues are partly covered by the linker not included in the constructs for individual domains. Peptides 331–339 (on the surface of the catalytic domain) and 454–473 and 474–488 (in the CWT, partly in regular β-structure, but also containing surface exposed loops and edge strand fragments) are not in contact with the other domain in any of the four molecules in the crystal. Changes in their exposure to solvent between domains and full-length enzyme therefore require domain arrangements not observed in the structure, or alternatively ‘interdomain communication’ (Fig. 7A).

The above observations suggest that lysostaphin may anchor itself to peptidoglycan via its CWT domain, which could then cleave different interpeptides in the region, thus locally weakening peptidoglycan in a more effective way than by random cleavages throughout the polymer. The precise number of *S. aureus* interpeptide cross-bridges is unfortunately difficult to estimate, because the peptidoglycan structure is uncertain and because ‘parallel stem peptide’ models predict closer packing of cross-bridges than ‘antiparallel stem peptide’ models. We note that a modular architecture of peptidoglycan hydrolases is fairly common 37–40, particularly in phage from Gram-positive bacteria, which also need to keep lysis local 41,42. The benefit from tethering a catalytic domain to a CWT domain is nicely illustrated by grafts of CWT domains to unrelated peptidoglycan hydrolases 43 and may at least in part explain the superiority of lysostaphin over LytM in *in vivo* applications.

## Experimental procedures

### Protein purification

The expression plasmid for lysostaphin (pET21) encoded the catalytic and CWT domains (residues 248–493, UniProt entry P10547) and a C-terminal His-tag (LEHHHHHH sequence) under the control of the T7 promoter and lac repressor, as described previously 44. BL21(DE3) *Escherichia coli* cells were grown in 2× YT media to a *D*_600_ of 0.6, induced with 1.2 mm isopropyl β-d-thiogalactoside, and grown for a further 3 h at 30 °C. The collected cells were harvested and the lysate was used for protein purification. The first purification step was performed on a Zn^2+^ column (5 mL Pharmacia HiTRAP chelating column charged with 50 mm ZnCl_2_). The imidazole gradient was run from 5 mm to 1 m in 20 mm sodium phosphate pH 7.0, 0.5 m NaCl. Histidine-tagged lysostaphin eluted at 0.3–0.4 m imidazole. Gel filtration on a Sephacryl S100 column in 20 mm Tris/HCl pH 7.0, 0.5 m NaCl, 0.2 m imidazole was done as the final step of protein purification. After gel filtration, the protein sample was dialyzed against 20 mm Tris/HCl pH 7.0, 50 mm NaCl and concentrated.

The lysostaphin catalytic domain (residues 251–384) was cloned into the *Nco*I/*Xho*I site of a pET15b plasmid. The 134 amino acid peptide was overproduced in BL21 (DE3) *E. coli* in Luria–Bertani media by induction with 0.1 mm isopropyl β-d-thiogalactoside at 25 °C for 6 h. The cell pellet was suspended in 20 mm Tris/HCl pH 7.0, 1 m NaCl, 10% glycerol and disrupted by Constant Cell Disruption System (Constant Systems Ltd, Daventry, UK). Cell lysate was dialyzed against 20 mm Tris/HCl, pH 7.0, 50 mm NaCl and purified on SP Sepharose column (GE Healthcare, Uppsala, Sweden). The NaCl gradient was applied and the protein was eluted at ∼ 0.9 m NaCl. Further purification was done by gel filtration on Superdex 75 column (GE Healthcare) in dialysis buffer.

### Crystallization

All crystallization experiments were carried out by the vapor diffusion method in sitting drops at 4 °C. Mature lysostaphin was concentrated to 8 mg·mL^−1^, and mixed in a 1 : 1 ratio with the reservoir buffer containing 0.1 m Mes/NaOH pH 6.5 and 1.6 m magnesium sulfate and 2 mm tetraglycine phosphinic acid. Drops were then equilibrated against the reservoir buffer. EDTA and ammonium sulfate as additives improved crystal quality. Prior to flash-cryocooling, the crystallization buffer was supplemented with 25% (v/v) glycerol. Lysostaphin crystals belonged to the cubic space group P4(3)32 and diffracted to 3.5–4 Å resolution at synchrotron beamlines.

Crystals of the catalytic domain of lysostaphin in the absence of phosphate were grown within a month by mixing an 11.5 mg·mL^−1^ solution of the protein in a 1 : 1 ratio with reservoir buffer Morpheus 2-46 (Molecular Dimensions, Newmarket, UK). This buffer is composed of 0.1 m Tris (base), Bicine pH 8.5, 0.1 m amino acids [l-Na-glutamate, alanine (racemic), glycine; lysine-HCl (racemic); serine (racemic)], 30% v/v precipitant (ethylene glycol, PEG 8000). Crystals grew during a month of equilibration against the reservoir buffer. Crystals were flash-cooled directly from the drop. Thirty minutes prior to setting up the crystallization trials, the protein had been supplemented with 6 mm of the pentapeptide GGSGG, which should be resistant to lysostaphin cleavage because of the presence of the serine residue. However, there was no electron density for the pentapeptide found in the crystal.

Crystals of the catalytic domain of lysostaphin in the presence of phosphate were obtained by mixing the protein solution at a concentration of 11.5 mg·mL^−1^ in a 1 : 1 ratio with the reservoir buffer Structure Screen 1–46 (Molecular Dimensions) containing 0.05 m potassium dihydrogen phosphate and 20% w/v PEG 8000. Similarly as in the absence of phosphates, the crystals grew within a month. Prior to flash-cryocooling, the crystallization buffer was supplemented with 10% (v/v) glycerol. Thirty minutes prior to setting up the crystallization trials, the protein had been supplemented with 6 mm glycine hydroxamate. Crystals were soaked for 5 min with 25 mm iodo-GGSGG pentapeptide dissolved in the buffer used for cryocooling, but no electron density for the putative inhibitors was observed.

### Structure determination

The crystals of the full-length lysostaphin were tested on multiple beamlines (of DIAMOND, ELETTRA, PETRA and BESSY) aiming at the highest possible data quality. The best dataset was collected on the MX 14.1 beamline of BESSY at a wavelength of 0.9184 Å. Two wavelength MAD data that have been used for phasing were collected on DIAMOND IO2 beamline to 4.0 Å. The two final datasets for the lysostaphin catalytic domain were collected at MX 14.1 (1.26 Å) and 14.2 (1.78 Å). All diffraction data were processed using the xds suite 45.

The full-length lysostaphin structure was solved by a combination of the molecular replacement (MR) and the MAD methods. Careful preparation of the search models proved crucial. Multiple structure-based sequence alignments were generated for the homologues of the catalytic and CWT domains of lysostaphin, using the DALI sever 46. The alignments revealed potential flexible loop regions (indicated by high rmsd values and high crystallographic temperature factors), which were deleted for the MR search. Finally, the structures of *S. aureus* LytM (PDB ID 2B0P, chain A 19) and *S. capitis* Ale-1 (1R77, chain A 20) were chosen as search models based on the low overall temperature factors and high resolution. A sequential MR search scheme was carried out using phaser 47. The first three copies of the truncated LytM search model were placed correctly with high log likelihood gain values. Placement of three copies of truncated Ale-1 in subsequent MR search steps raised the log likelihood gain value of the solution to over 1000. Placement of further molecules by MR failed. Therefore, a two-wavelength MAD experiment (peak wavelength 1.2827 Å and inflection wavelength 1.2831 Å) was performed to obtain experimental phases. Zn^2+^-binding sites were located in the anomalous difference Fourier map calculated with peak wavelength diffraction data and model phases. Altogether, only four Zn^2+^ ions were found, strongly suggesting that the crystals contained four molecules of lysostaphin in the asymmetric unit. The programs sharp 48 and solomon 49 were used to calculate an experimental electron-density map, which was very clear even without noncrystallographic symmetry averaging. The two missing domains (one catalytic and one CWT) were tentatively built by manual density interpretation. Electron density was then cut out in their vicinity and used for additional real-space molrep 50 searches using the models of the catalytic and CWT domains. The linkers between cell wall and catalytic domain were built manually with the coot program 51. Register errors are very unlikely in the globular domains and the better defined parts of the linkers. In all four cases, the electron density is of sufficient quality to decide with confidence which catalytic and CWT domains are covalently linked. In the most ill-defined regions of the linkers, Ramachandran indicators suggest that the model is poorer than elsewhere, and we cannot exclude register errors. Refinement of the mature lysostaphin was carried out in refmac 52 with separate noncrystallographic symmetry restraints for CWT (residues 403–493) and catalytic (residues 278–385) domains, but no noncrystallographic symmetry restraints for the linkers (386–402). The model was refined to the final *R*-factors of *R*_cryst_ = 27.8% and *R*_free_ = 29.6%. Geometry indicators of the model were typical of structures at this resolution (rmsd bond lengths = 0.012 Å, rmsd angles = 1.4°, 99.3% of residues in the allowed region of the Ramachandran plot).

The crystals of the lysostaphin catalytic domain had a nearly orthorhombic lattice. However, scaling of the diffraction data indicated that the crystals were only monoclinic. Extinctions on the axis of symmetry identified the space group as P2(1). An analysis of the solvent content suggested the presence of two catalytic domains in the crystallographic asymmetric unit. The crystal grown in the presence of phosphate showed clear indications of twinning and thus test reflections were selected in thin resolution shells. The structure was solved by MR with the help of the catalytic domain of the mature enzyme. The model was rebuilt automatically with the arp/warp program 53. The structure was refined with phenix 54 in the twin refinement mode, which reduced the R factors by ∼ 7%. The crystal grown in the absence of phosphate was solved with MR using the refined catalytic domain structure as a model. The test reflections were chosen consistently. The crystal was did not appear to be significantly twinned, as judged by xtriage 54, therefore the refinement was carried out in the standard mode in refmac 52. Data collection and refinement statistics are presented in Table 1. The atomic coordinates and corresponding structure factors have been deposited at PDB with the following accession codes: full-length lysostaphin, 4LXC; catalytic domain in the presence of the phosphate, 4QP5; and in its absence, 4PQB.

## Abbreviations

CWT: cell-wall-targeting domain
MAD: multiple anomalous diffraction
MR: molecular replacement
